# Impact of Light Environment on Driver's Physiology and Psychology in Interior Zone of Long Tunnel

**DOI:** 10.3389/fpubh.2022.842750

**Published:** 2022-03-03

**Authors:** Li Peng, Ji Weng, Yi Yang, Huaiwei Wen

**Affiliations:** School of Architecture and Urban Planning, Chongqing University, Chongqing, China

**Keywords:** interior zone, long tunnel, heart rate variability, perceived ambient luminance, physiological fatigue, mental stress

## Abstract

In tunnels, lighting not only affects visual performance, but also non-visual aspects such as drivers' physiological fatigue and mental stress. The non-visual impacts in the interior zone of long tunnels are particularly prominent as drivers are confined for a long time. To alleviate this problem, this study aims to investigate the relationship between drivers' physiological and psychological states and lighting environments. The physiological signal test system (MP150) breathing belt was used to record the changes of heart rate variability (HRV) of drivers when passing through the interior zone of a long tunnel under various lighting conditions. In particular, sympathetic indicators of physiological fatigues and the ratio of low frequency and high frequency (LF/HF) representing mental load were obtained. By analyzing the temporal variation in these two indicators, it is found that environmental luminance perception can more accurately reflect drivers' physiological and psychological states in the long tunnel than road luminance. An increase in road luminance or background luminance will result in a decrease in the mental stress, thereby reducing fatigue sense. Compared to simply increasing road luminance, mental stress of drivers decreased more obviously when the background luminance of long tunnel increased. Based on this, this paper proposed a method to regulate non-visual effect by adding contour markers without increasing light source intensity for the improvement in lighting performance, driving safety, and energy efficiency in long tunnels.

## Introduction

In long tunnels, reasonable lighting design is one of the important tasks for engineers and designers to ensure driving safety. Traditional tunnel lighting studies have paid attention to the driver's luminance adaptation, to address the luminance problem at the entrance and exit of the tunnel. However, for long tunnels, existing studies are far from enough to solve these problems. Meanwhile, existing technical reports and specifications have not particularly presented technical indicators for long tunnels. There is a gap between light performance and driving comfort and safety in the interior zone in long tunnels. According to the Statistics of Road Tunnel Traffic Accidents in China from 2012 to 2016 (1), the number of casualties caused by tunnel accidents in 2016 increased by 25% compared with 2012. Most accidents are related to the rear-end collision of vehicles in tunnels.

In the interior zone of long tunnels, due to the low luminance of the road, non-visual impacts such as driver's physiological rhythm and psychological state change, making drivers prone to fatigue and sleepiness, which potentially leads to accidents. Meanwhile, the driver's operation can be potentially reduced, where the amount of visual information can be reduced by about two-thirds compared with ordinary road sections, which leads to fatigue-related distraction (2, 3).

The research on lighting environment has long been carried out based on the human visual pathway. It has been established to meet the requirements of illuminance level and visual recognition, cognitive efficiency, color rendering of light source, and glare evaluation methods (4, 5). However, research in the past 20 years has shown that lighting not only directly affects people's visual system, but also physiology and psychology. In 2002, Berson et al. (6) from Brown University discovered the third photoreceptor cell “retinal ganglion cell” (RGC) and verified its non-visual effects, started research on the effects of light on human physiology. Pier-re Philip (7) investigated the effect of fatigue on driving behavior by comparing the normal group and the sleep-deprived group. The results showed that fatigue had a significant impact on driver's reaction time. Forsman et al. (8) studied driver's moderate fatigue and concluded that steering wheel variability data and lane offset variability data can be used as the characteristic parameters of fatigue. Yamaguchi et al. (9) used a hand-held fatigue detection system to evaluate the driver's sympathetic excitement through the activity of salivary enzymes and concluded that the decrease in sympathetic excitement means that the driver is immediately fatigued. de Naurois et al. (10) found that some subjects reached sleepy state after 10 min of driving in simulation test, and this time would be shorter in real driving. Wang et al. (11) established a calculation model of driving fatigue index based on fuzzy mathematical evaluation by simulating the change rules of dependent variables such as EEG, ECG, eye movement, and driving performance. Although neuroimaging studies have shown that both the prefrontal cortex and parietal lobe have light-induced activities related to visual-spatial ability and executive function (12), the blue-rich light has only partially explored these processes. Exposure to high luminance will preferentially reduce reaction time, sleepiness, and auditory failure and improve alertness (13). There is growing evidence that the spectrum and luminance environment can have a strong impact on visual task performance, safety, and comfort (14, 15). It should be noted that lighting choices increasingly inclined to profit from non-visual impacts (16).

Tunnel lighting also strongly affects human psychology. Psychologically, a lot of repetition in a short time is prone to boredom and reduces sensitivity to repetitive stimulation. The repeated dim visual environment in tunnel makes the driver easy to fall into “tunnel hypnosis” state, reduce concentration and alertness, which causes serious hidden dangers. Meanwhile, the decrease of alertness will further aggravate physiological fatigue. Some scholars explained the relationship between the increase in physical fatigue and the increase in mental load in non-visual impacts from the aspect of driving workload (17, 18). In recent years, researchers have found that drivers need enough visual stimulation to keep their nerve activity excited and maintain sufficient alertness. So, they attempted to improve the sidewalls and contour markings in the tunnel to improve driver's comfort (19). Min et al. (20) analyzed the natural responses of young subjects under different speeds and driving modes by testing their EMG, heart rate, and skin temperature. The results showed that mental load increased with the increase of speed, and it increased significantly when stopped or accelerated. Craig and Tran (21) studied the relationship between various psychological signals and driving fatigue by recording the blinking phenomenon and face color of subjects and pointed out that psychological factors are related to self-evaluation. Besides, some scholars focus on the influence of spectrum and color temperature on alertness, job performance, and work efficiency (12, 22). For the illumination sources with high relative spectral value in the shortwave direction, illumination can strongly improve alertness, reduce reaction time, and subjective sleepiness and improve cognitive performance for tasks requiring concentration (23–25). It solves the problem of driving and subsequent sleepiness under low color temperature light sources (8). The study also found that the above phenomena are particularly obvious under continuous illumination, especially under short wavelengths around 460 nm (13, 23). The above research fully shows the relevant factors in tunnel lighting not only improve driver's physiological fatigue, but also reduce mental stress and reaction time to obstacles, and improve the driving comfort. At the same time, the reaction time of the driver facing obstacles in the tunnel is reduced and the driving comfort is improved (26, 27). These factors include road luminance, sidewall luminance, color temperature, and relative spectral values in the shortwave direction, etc.

Although previous studies have focused on the non-visual impacts of lighting in the interior zone of long tunnels, there is a lack of research on ways to improve light environment. In recent years, tunnel lighting designers have begun to focus on this field by changing part of the lighting environment in the interior zone to improve the problem of reduced driver's alertness for long-term driving (28). Some have adopted the method of setting special light bands in the interior zone (29), some have used the effect of surface pavement materials to facilitate mutual reflection of light (30), and some have studied the impact of improving the reflectivity of tunnel sidewall materials (31) and improving safety aids in tunnels (32) on driving safety. In fact, as the mechanism has not been perfected, this research only focuses on the impact of light on human visual load. There are studies on the changes in the light environment in tunnel to relieve driver's fatigue (29), but it is not clear that the range of light and the luminance of light belt setting area have different degrees of fatigue relief. To sum up, present understanding of photobiological effect is limited, and the evaluation methods for driver fatigue are mainly divided into subjective evaluation and objective evaluation. Subjective evaluation methods mainly adopt questionnaires or evaluation scales, such as Karolinska Sleep Scale (KSS) and task load index, which can directly reflect driver's physical fatigue and mental load. However, subjective evaluation is greatly affected by individual differences and is highly subjective, so it is often used for qualitative comparisons. Objective methods for detecting driver fatigue are numerous, which often include physiological measurements (33) (mainly including brain and heart activity) and body measurements (34) (mainly including eyelid movement, head movement, and facial expression). However, the collection of objective physical data has corresponding various conditions and is more affected by an individual. Therefore, mathematical models should be skillfully used to eliminate individual influences and analyze objective laws when processing data. This study uses sympathetic nerve activity (SA) and low frequency and high frequency (LF/HF) separated from HRV data in electrocardio (EEG) by software as dependent variables to observe and analyze drivers in different intermediate luminance environments. Additionally, the study aims to investigate as follows:

1. Changes in driver's physical fatigue and mental load in long tunnel with low perceived ambient luminance;
2. Driver's physical fatigue and mental load characteristics under the condition of changing road luminance or changing perceived environment luminance;
3. The reason for reducing physical fatigue and alleviating mental load after adding contour section.

## Methods

### The Variables Selected by the Experiment

**Figure 1:**
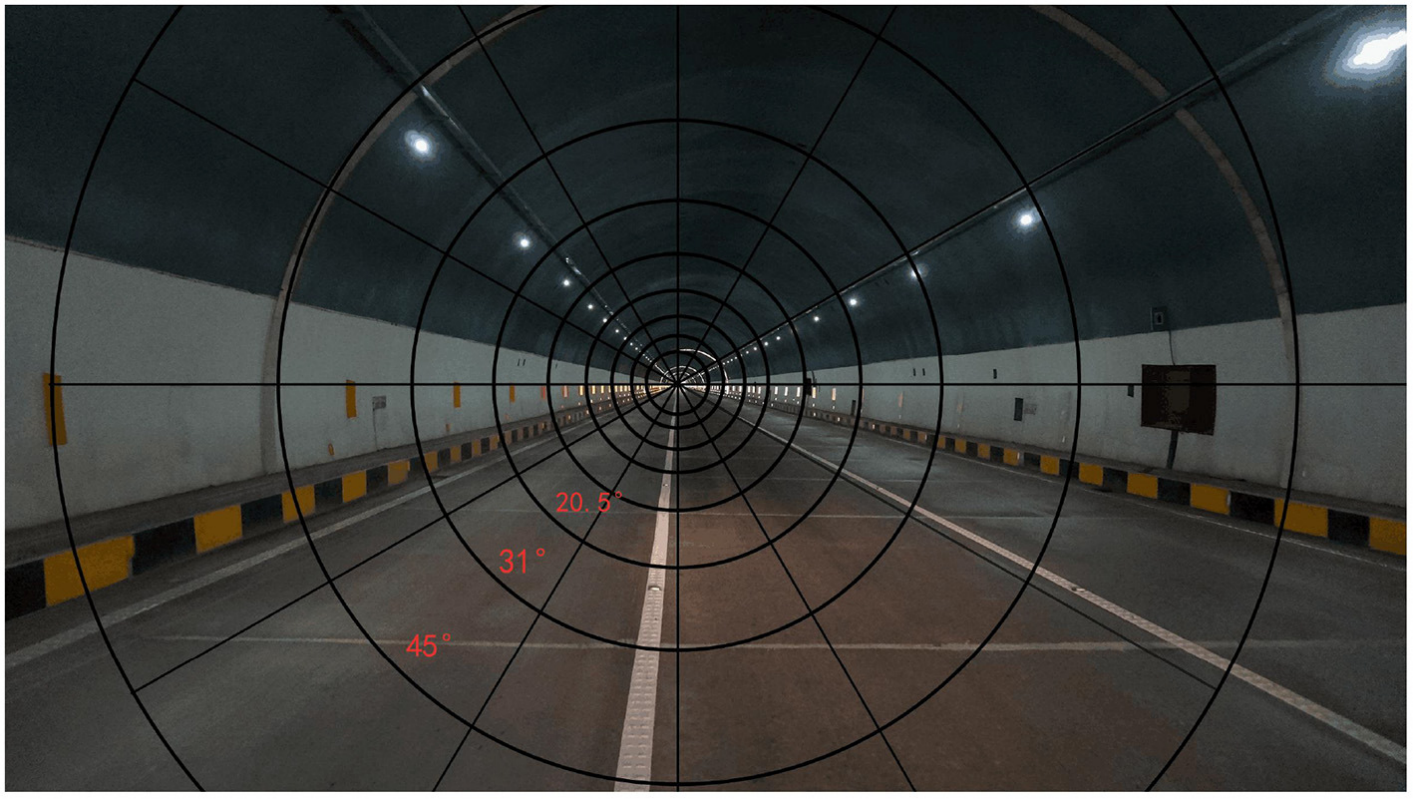
The driver's range of perception at different speeds.

In the long tunnel driving behavior, the driving safety is not only affected by the road luminance, but also the overall environmental lighting level in the driver's field of view including the road. Under the condition of the same road luminance, drivers' visual perception and non-visual effects will be completely different due to the difference of environmental luminance and color temperature in the viewing area. In a long tunnel, when the driving speed is too fast, the driver's visual area will be significantly reduced and has an obvious inverse relationship with the speed. In this paper, the average luminance within the visual area is defined as the perceived ambient luminance, which includes the road luminance and the luminance reflected from the sidewall of the tunnel into the human eye under the irradiation of the headlights. The specific calculation of the perceived environmental luminance under a certain speed can be based on the relationship between Thomas's perceived range and the speed. For example, when the driving speed is 80 km/h, the background luminance norm perceived by the driver in the dynamic field of view is the cross-section in the range of 31° with the long tunnel as the center. Figure 1 shows the corresponding range of perceived ambient luminance at different speeds. The perceived ambient luminance is different from the overall background luminance, which emphasizes the visual perception range of the driver. However, it is difficult to obtain the perceived ambient luminance accurately. The maximum field angle of traditional point-of-view luminance meter is only 1, which makes it difficult to measure and calculate the perceived ambient luminance in such a large range. Thanks to the development of an equivalent screen luminance meter, the ambient luminance in polar coordinates can be measured. In this paper, pavement luminance and perceived environment luminance are considered as independent variables.

In the actual operation of the tunnel, adding lighting can significantly improve the luminance of the road surface and the perceived ambient luminance. In consideration of energy-saving and traffic flow and other relevant factors, the road luminance in the interior zone is regulated in CIE technical reports and other countries. According to Chinese Guidelines JTG/TD70/2-01-2014 (35), the road luminance value in the interior zone of the tunnel is determined according to the design speed, traffic flow, and other factors, as shown in Table 1.

**Table 1:** JTG/T D70/2-01-2014 about the value of road luminance in the interior zone of tunnel.

**Design speed (km/h)**	**Traffic volume**	**Traffic volume**	**Traffic volume**
	***N* ≥650*veh* /(*h In*)**	**180*veh* /(*h In*) < N <650*veh* /(*h In*)**	***N* ≥180*veh* /(*h In*)**
120	10 cd/m^2^	6 cd/m^2^	4.5 cd/m^2^
100	6.5 cd/m^2^	4.5 cd/m^2^	3.0 cd/m^2^
80	3.5 cd/m^2^	2.5 cd/m^2^	1.5 cd/m^2^

According to the content in Table 1 and different driving speeds, 3.5, 6.5, and 10 cd/m^2^ were selected as road luminance under the condition of maximum traffic flow and analyzed as independent variables of the unset profile markers. Contour markers with different densities were set at the lowest luminance (3.5 cd/m^2^) and included in the comparison group.

Electroencephalography (EEG) and electrocardiogram (ECG) are the most common indicators of physiological and psychological changes in the human body, and ECG is more appropriate when the subject is under stress. In ECG measurements, a person's heart rate measures the number of beats per minute. But most of the time, the heart rate stays in a very stable range and there is no significant difference. Therefore, the dependent variable involved in this study is not ECG or heart rate directly, but heart rate variability (HRV), which reflects the difference between adjacent R-R intervals, which is an indicator that can accurately reflect the real physiological and psychological conditions of drivers under driving pressure. In practice, the continuously measured ECG waveform is usually used to directly calculate and analyze the discrete degree of the corresponding heartbeat interval. In HRV, a large number of frequency domain and time domain indexes can be separated, and the related indexes in the time domain and frequency domain are often used as indexes to analyze the physiological and psychological changes of the subjects. For example, standard deviation of NN (SDNN) intervals is used to analyze ECG cycle variation by calculating the standard deviation of 24-h normal heartbeat interval. Generally speaking, the younger the age, the greater the value of SDNN. Number of pairs of NNs that differ by more than 50 ms (NN50), the number of heartbeats in an ECG that differ by more than 50 ms between normal adjacent beats, is used to evaluate a person's state of emotional tension. Sympathetic nerve activity (SA) can directly evaluate the degree of excitement. Low-frequency (LF) power or high-frequency (HF) power represents the balance degree of autonomic nerve activity.

In this paper, indicators of physiological fatigue and psychological load were selected based on the literature (36, 37), SA was used as an indicator of physiological fatigue, LF/HF ratio was used as an indicator of psychological load, and a mathematical model was used to conduct a comprehensive score analysis of the two indicators.

### Setting of Experimental Conditions

**Figure 2:**
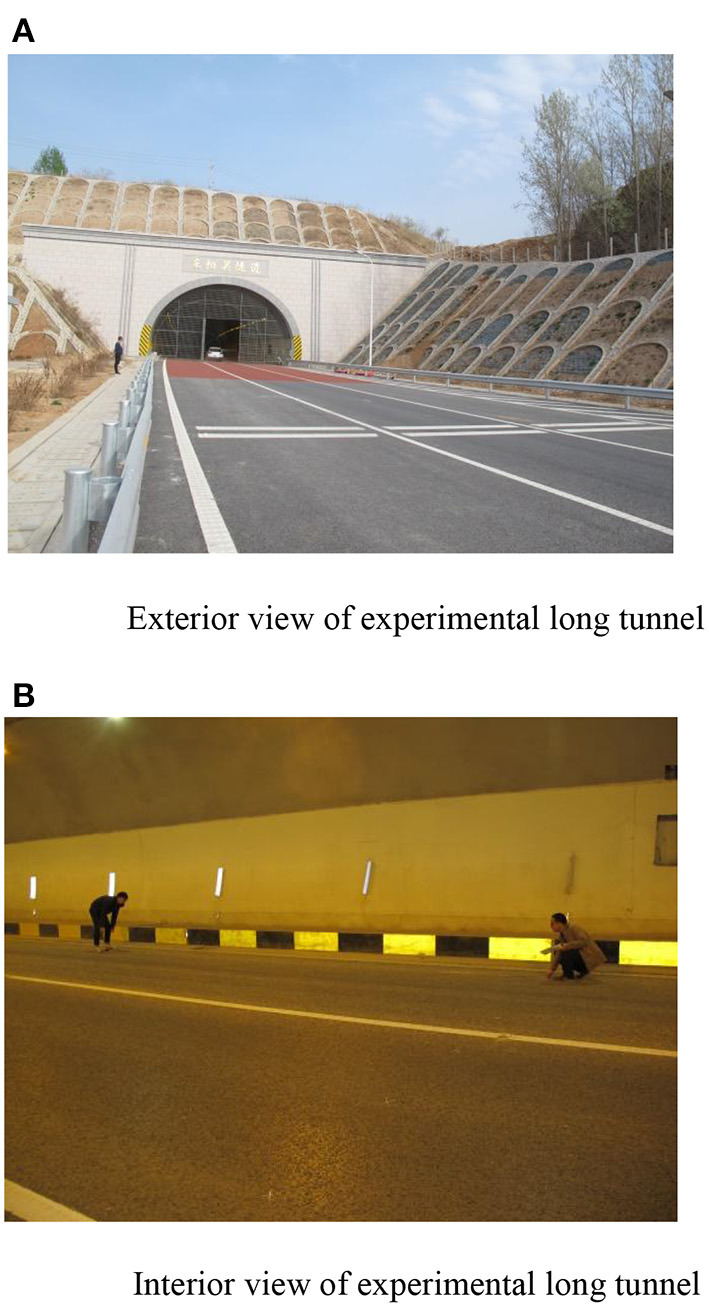
**(A)** Exterior view of experimental long tunnel. **(B)** Interior view of experimental long tunnel.

To get closer to the real driving situation, this paper chose Dongyangguan Tunnel in Changzhi City, Shanxi Province, China (Figures 2A,B) for field test. Dongyangguan tunnel is 8,360 m long. According to the setting requirements of the test section, the research group selected the interior zone of the tunnel about 6,300 m for round-trip driving, and the test distance was 12,600 m in total. The lighting arrangement of the tunnel is symmetrical lighting arrangement. In this paper, two different types of experimental groups were set up in the inner area of the long tunnel. One is to control the experimental conditions of ground luminance by stepless dimming of LED without contour mark. According to the provisions of Table 1, 3.5 cd/m^2^ (experimental group A), 6.5 cd/m^2^ (experimental group B), and 10 cd/m2 (experimental group C) were selected as road luminance for the experiment. In the other category, contour markers with 8-m spacing (experimental group D) and 4-m spacing (experimental group E) were set on the latter half of low-luminance road surface (3.5 cd/m^2^), and the length of contour markers was 6,300 m, 1.2 m above the ground. The details of the five experimental groups set in this paper are shown in Table 2. To accurately measure the road luminance and the perceived ambient luminance under vehicle lights, a PR-920 light screen luminance meter was used to measure the different road brightness and perceived ambient luminance.

**Table 2:** Five different experimental conditions were set in the experiment.

**Experimental condition number**	**Set mode**	**Road surface luminance (cd/m^**2**^)**	**Perceiving ambient luminance (cd/m^**2**^)**
A	Reverse driving of the front part + forward driving of the back part after occlusion contour mark	3.5	2.71
B	Reverse driving of the front part + forward driving of the back part after occlusion contour mark	6.5	3.52
C	Reverse driving of the front part + forward driving of the back part after occlusion contour mark	10	3.85
D	Reverse driving for the front part + forward driving for the back part with 8-m spacing outline mark	3.5	3.54
E	Reverse driving for the front part + forward driving for the back part with 4-m spacing outline mark	3.5	4.51

To explore the difference between simulated driving and actual driving, the research group selected Dongyangguan Tunnel in Changzhi City, Shanxi Province, China for field test. In addition, the interior zone of the tunnel was selected as the test section, which was driven at 80 km per hour for 10 min with cruise control enabled.

### The Experiment Equipment and Subjects

The main instruments used in this experiment are PR-920 light screen luminance meter and MP150 multichannel physiological signal meter. The testing process of PR-920 screen luminance meter. The characteristic of this luminance meter is that it can measure the luminance of the screen through the captured picture information, which avoids the shortcoming of too small field angle of traditional luminance meter. Specifically, in the case of low luminance, the imaging luminance meter can be multiple sampling overlay, which makes the measurement more accurate.

The physiological monitoring instrument is MP150 multiguide physiological signal instrument and supporting breathing band produced by Biopac Company. This type of breathing band is suitable for outdoor real-time measurement of respiratory rate, heart rate, and HRV, and the data obtained are accurate and easy to use.

The driver's heart rate data are recorded in Biopac's portable breathing strap, which is connected to the computer *via* Bluetooth. Two cameras are installed in the car to ensure that the driver maintains a fixed sitting posture. The measurement characteristics are as follows:

1. Sampling frequency: 50 Hz, system delay <50 ms;
2. Acquisition parameters: real-time ECG;

The vehicle used in the experiment is GAC Mitsubishi SUV (made in China, capacity: 2.5 L). Auxiliary devices are voltage regulators and personal computers.

Gender, age, and driving age all affect the accuracy of HRV measurement. A total of 10 healthy male volunteers were enrolled with an average age of 30.6 years (standard deviation: 2.2). Each driver had a valid license corresponding to the test vehicle, and the average driving experience of the drivers was 9.6 years (standard deviation: 3.9). They had normal hearing and normal vision or corrected vision. All subjects are proficient in driving this type of vehicle without serious accident experience, and the subjects are healthy without physical or physical defects, diseases, or sleep disorders. They were also asked to avoid alcohol, caffeine, and drugs for 24 h before the test and to maintain a normal sleep schedule for at least eight h a night before the test. Participants entered the test after giving informed consent for their physiological information to be collected.

### The Experiment Procedure

Two h before the experiment, subjects were required to undergo a 2-h long highway driving process and then complete the assembly of the portable breathing belt of Biopac. The tester puts on the MP150 portable physiological signal measurement system for the driver and informed the test task. The subjects sit in the driver's seat of the car in a comfortable posture and keep a visual posture in front. The tester sits in the passenger seat of the car and connects the sensor on the bandage to the computer through Bluetooth function. AcqKnowledge 5.0 software is used for real-time monitoring and recording. Physiological data of subjects in initial condition (HRV) shall be recorded before the test begins. After that, the experimental conditions of lighting in the tunnel were adjusted to experimental condition A as shown in Table 2. Subjects were asked to start the car at the entrance section of the tunnel and ensure that the driving speed of the car could reach the designed driving speed of 80 km/h before entering the interior zone of the tunnel. Due to the limitation of tunnel length, the subjects entered the tunnel in reverse direction first, turned around immediately at the end of the first half of the experiment, and then drove to the starting point. When starting the car, the subjects started the software system and began to record their original heart rate indicators. After all drivers complete a lighting test, adjust the lighting mode in the tunnel to test conditions B, C, D, and E and repeat the above test process. The order of subjects was shuffled throughout the experiment to avoid differences caused by the order of conditions.

According to the different experimental conditions set, the original data collected by the MP150 physiological system are processed as follows:

1. The original ECG data were exported and filtered to calculate the HRV using AcqKnowledge 5.0 software.
2. The initial time interval of HRV data processing was set as 30 s, and all time domain and frequency domain indicators of each subject were averaged within 30 s.
3. The sympathetic nerve activity index SA and the ratio of LF/HF were selected from the processed time domain and frequency domain indexes.
4. The statistical significance of SA and LF/HF was explained.

## Results

By conducting 10 tests for each experimental condition (1 test for each of 10 subjects), 180,000 sets of data including SA and LF/HF were obtained during the experiment, and the data that deviated significantly from the fluctuation range were excluded. After that, the data are condensed every 30 s to obtain 6,000 sets of data. Analysis of variance (ANOVA) was used to analyze the reliability of the data, which includes three sources of variation, LF, HF, and SA. The results of variance analysis are shown in Table 3. To analyze the significance of physiological indexes at different sections of the tunnel, the normal distribution test was carried out for these 6,000 groups of data. The results show that after Shapiro–Wilk test, the data of each group accord with normal distribution. According to the above results, the ratio of SA to LF/HF separated by HRV has a significant difference in different experimental groups in the interior zone of long tunnel.

**Table 3:** Test results.

**Experimental conditions**	**HRV**								
	**The low-frequency energy**			**The high-frequency energy**			**Sympathetic indicators**		
	**The average**	**F**	**P**	**The average**	**F**	**P**	**The average**	**F**	**P**
A	0.4377	7.6	<0.001^***^	0.1863	2.5	0.055^*^	0.2811	8.5	<0.001^***^
B	0.4819			0.2032			0.2963		
C	0.8262			0.3499			0.2981		
D	0.4136			0.1745			0.2953		
E	0.8372			0.3513			0.3051		

* *means relevant*;

*** *means very relevant*.

### HRV Changes of Subjects Without Contour Markers (Experimental Conditions A, B, C)

**Figure 3:**
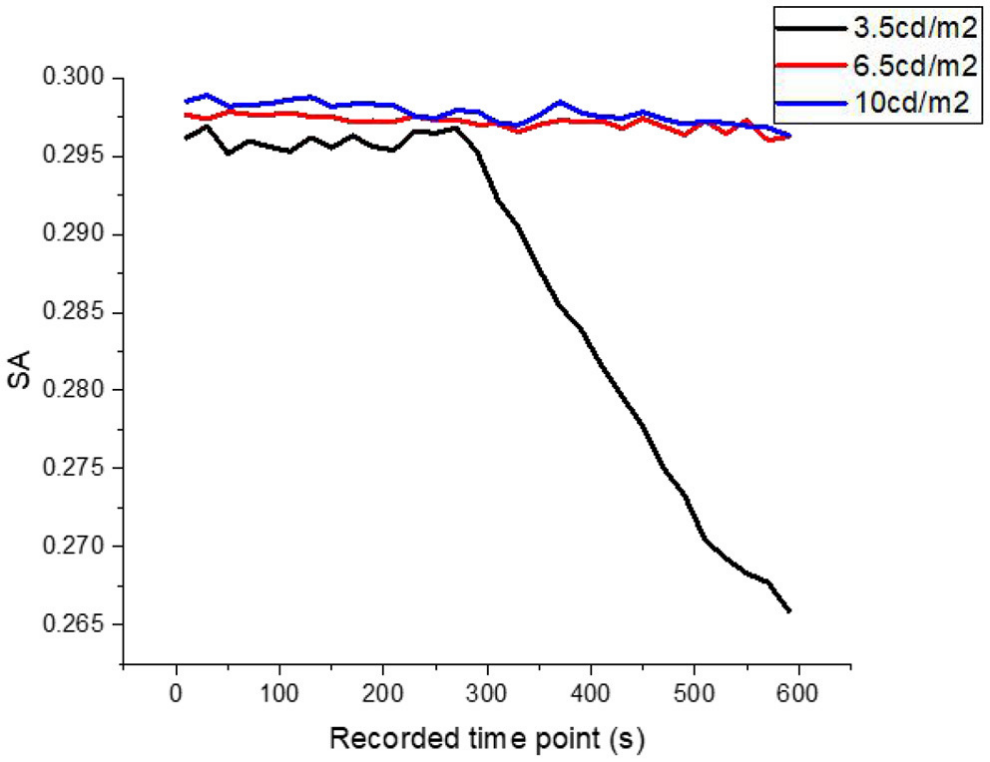
When the road luminance is 3.5, 6.5, and 10 cd/m^2^, the variation trend of average SA of the subjects.

**Figure 4:**
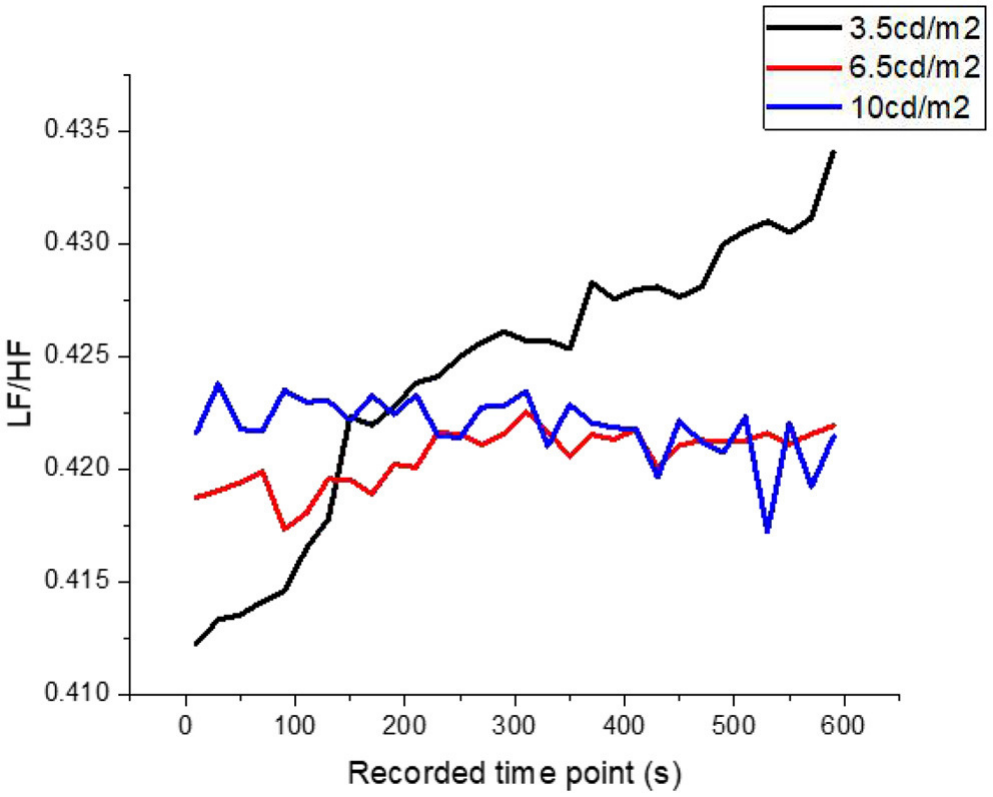
When the road luminance is 3.5, 6.5, and 10 cd/m^2^, the variation trend of average LF/HF ratio of subjects.

Under the condition that road luminance is only changed without contour markers, the average SA and LF/HF ratio in HRV of the tested driver change with driving time, as shown in Figure 3. The average value of the LF/HF ratio in the HRV of the tested driver varies with the driving time, as shown in Figure 4.

As can be seen from Figures 3, 4, when the road luminance level in the tunnel is low luminance (3.5 cd/m^2^), the perceived environmental luminance is also 2.71 cd/m2, the lowest in the experiment setting. With the increase in driving time, SA and LF/HF of the tested show significant changes. These changes are manifested as follows: 1. SA decreased slowly from 5 min before to sharply after 5 min; 2. LF/HF rose rapidly from the beginning of the test. In this test, the average SA of the test subjects changed relatively slowly in the first 5 min, fluctuating around 0.295, but decreased rapidly from the 6th min until 0.265 at the end of the test, which indicates that the accumulated rate of fatigue of the test drivers was different. At the same time, the average LF/HF of the subjects reflected the continuous increase of the psychological stress they were under during the test. SA and LF/HF have a completely different variation trend under low road luminance, which rises from 0.412 to 0.435 at the beginning of the test. When the road luminance in the long tunnel area changed to medium luminance (6.5 cd/m^2^), and the ambient luminance changed to 3.52 cd/m^2^, the sympathetic activity index (SA) of the tested drivers fluctuated in the range of 0.2960–0.2980 and showed a significant downward trend as time went on. But it was 0.2963 at the endpoint of the final experiment. This value is larger than SA in the case of low road luminance. The LF/HF trend reflecting psychological load is similar to 1/SA, which can be regarded as the same accumulation rate of psychological load and physical fatigue under the condition of moderate road luminance. The physical fatigue and psychological load of the tested drivers are more influenced by individual factors such as age, driving experience, and adaptation to closed space.

When the road luminance of long tunnel continues to rise to 10 cd/m^2^, the perceived environmental luminance becomes 3.85 cd/m^2^, SA decreases from 0.2985 to 0.2970, and LF/HF fluctuates between 0.421 and 0.422. Through SA and LF/HF data, it was found that the accumulation rate of physical fatigue and mental load slowed down, and the accumulation rate of mental load was more gentle. The results indicate that the fatigue and psychological load of the subjects will not decrease significantly with the increase of road luminance when the driver is at high luminance level, and the physiological fatigue plays a dominant role over time. In actual tunnel operation, the increase of road luminance does not have a simple linear relationship with the reduction of physiological fatigue and psychological load of drivers. The setting of pavement luminance should not be as high as possible. Increasing the level of pavement luminance will cause waste of energy consumption.

### The HRV Changes of Subjects After Adding Contour Marking

**Figure 5:**
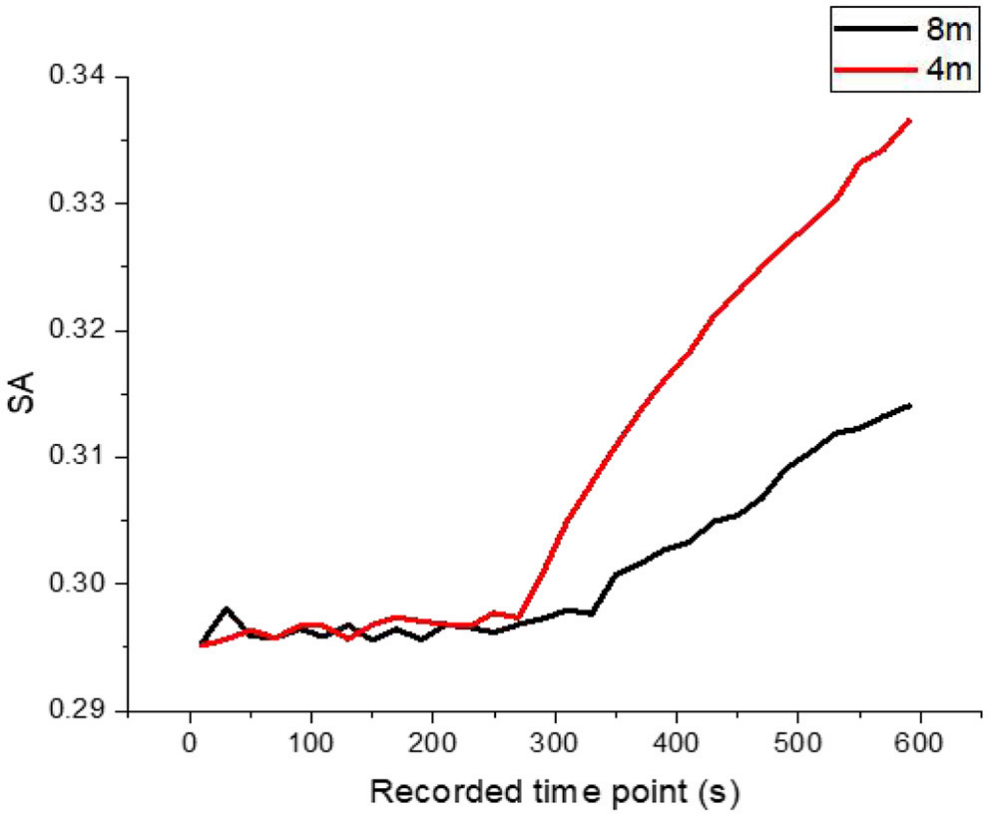
The variation trend of SA in subjects' HRV over time when using square delineators with 8 and 4-m spacing.

**Figure 6:**
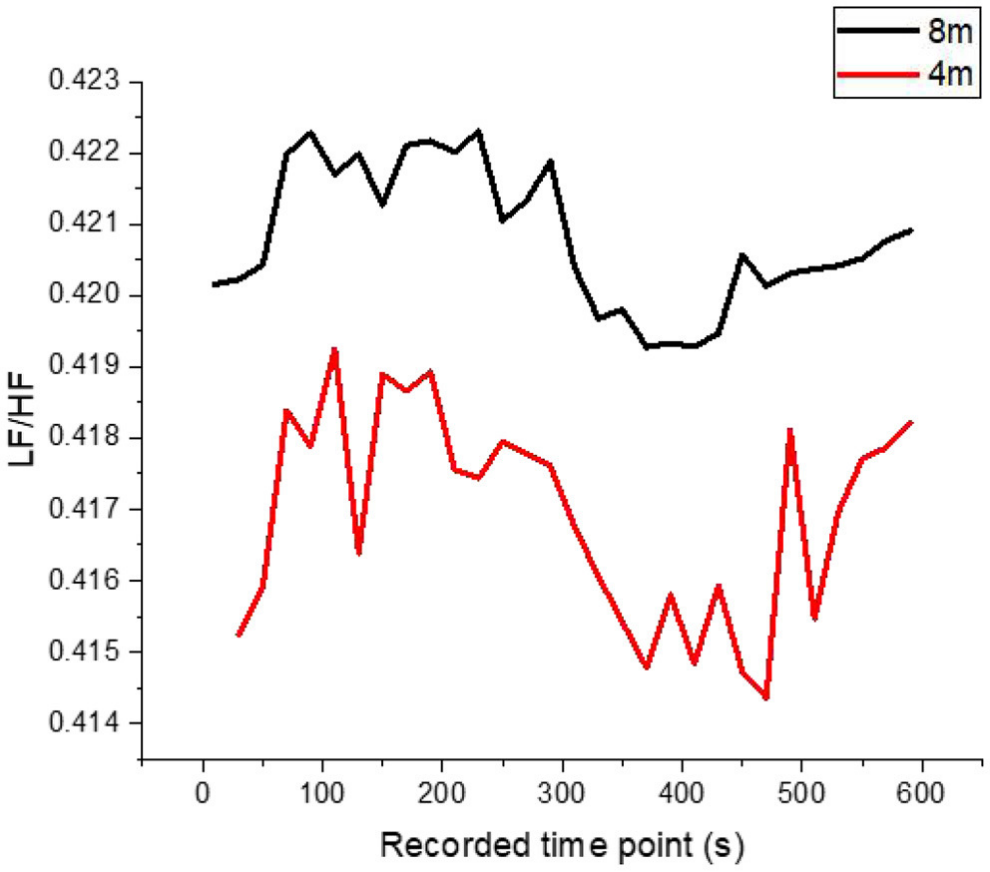
The variation trend of LF/HF ratio in subjects' HRV over time when using square delineator with 8 and 4-m spacing.

In the contrast group after the addition of contour markers, the road luminance was kept unchanged at 3.5 cd/m^2^. Contour markers with a spacing of 4 and 8 m were added in experimental conditions D and E, respectively, in the latter half of the journey. Due to the influence of headlights, the perceived ambient brightness of the tested drivers was significantly increased to 3.54 and 4.51 cd/m^2^. Under such experimental conditions, the relationship between SA and LF/HF in the tested drivers over time is shown in Figures 5, 6.

It can be seen from the experimental results that the road luminance of 3.5 cd/m2 is similar to that of the driver in the half distance without contour mark. SA fluctuated in a small range near 0.295, and LF/HF fluctuated between 0.416 and 0.420. The ratio of SA and LF/HF changed significantly during the half period when the contour marker was set. The variation trend of SA shows that the physiological fatigue degree of drivers decreases, and the increase of SA increases with the increase of the setting density of contour markers (from 8-m spacing to 4-m spacing) and finally increased from 0.315 to 0.325 at the end of the test, an increase of 3.17%. However, the LF/HF ratio, which reflects the psychological load, only decreased from 0.4210 to 0.420, decreasing by 0.24%. This indicates that in the range of subjects' perception, environmental luminance is one of the obvious stimulus factors. The greater the increment of environmental luminance, the faster the feedback of SA, the faster the degree of sympathetic nerve excitation, and the higher the upper limit of physiological fatigue improvement. The psychological load of drivers decreased after the setting of contour markers, but the degree of psychological load of drivers did not decrease obviously with the increase of setting density. It indicates that psychological load is associated with physiological fatigue degree of drivers to a certain extent, but SA and LF/HF ratio is still a relatively independent indicator, and the increase of fatigue degree of drivers (physiological aspect) does not affect the increase of psychological load (psychological aspect) to the same extent.

### Fuzzy Evaluation Model for Synthetic Fatigue

The existing evaluation methods mainly include regression analysis, factor analysis, gray relational analysis, and fuzzy comprehensive analysis. Fuzzy comprehensive analysis aims to quantify some evaluation objects with unclear boundaries and difficult to quantify, and it is suitable for the overall evaluation of evaluation objects with different attributes or affected by different indicators. Therefore, this paper adopts fuzzy comprehensive analysis to analyze and evaluate the driver's physiological indicators. Referring to the commonly used classification method in fuzzy mathematics (38), using k-means clustering analysis to cluster sample data of different indicators, the value ranges of different levels can be obtained:

- After calculating the distance between all samples and each center point, assign the samples to the corresponding category of the nearest center point.
- After reassigning the categories, calculate the corresponding center point of each category, as shown in Equation (1).
  (1) $$\begin{array}{l} S_{i}=\frac{\sum_{i=1}^{N}P_{i}}{N} \end{array}$$
  In Formula (1), *P*_*i*_ is the ith cluster center after reclustering; N is the number of samples in the category corresponding to the cluster center.
- Repeat steps 1 and 2 several times until the end of the iteration. The value range of evaluation grade of each measurement index can be obtained, as shown in Table 4.

**Table 4:** The value range of evaluation factors.

**Value range**	**Level 1**	**Level 2**	**Level 3**	**Level 4**	**Level 5**
SA	0.2972~0.3050	0.2935~0.2972	0.2877~0.2934	0.2748~0.2867	0.2119~0.2701
LF/HF	0.2689~0.3699	0.3790~0.4020	0.4038~0.4153	0.4154~0.4231	0.4231~0.4388

Based on the evaluation principle of fuzzy mathematics, this paper refers to the determination method of membership function in Zhou (35). For m factors, after calculating membership degree, if {ri1, RI2, RI3... Rin} is set as the I row, so a fuzzy matrix R0 which integrates M evaluation factors and N evaluation levels is formed and the membership degree is calculated as follows: Assume that the status value of the evaluation index is λ I, and the allowable range of this value is [API, BPI], and [AIJ, BIj] is the value interval of each evaluation grade. For each set of evaluation factors uij (I = 1,2..., m), the evaluation grade set vj (j = 1,2..., n) membership degree rij, the value of rij can be obtained in Formula 2.


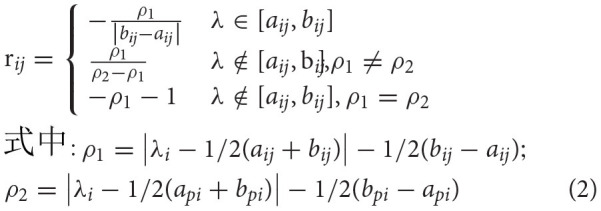


where RIj is the membership degree of evaluation index Ui corresponding to different grades of Vj. λ I is the average value of the sample data of the evaluation index, and its value range is [API, BPI]. API is the minimum value of the evaluation index, and BPI is the maximum value of the evaluation index. The value range of each evaluation grade corresponding to the evaluation index is [AIJ, BIj], where AIJ is the minimum value of the evaluation grade and BIj is the maximum value of the evaluation grade. By calculating the membership function, the membership matrix of each index corresponding to each level is obtained. The membership matrix of SA and LF/HF of subjects is shown in Table 5.

**Table 5:** Membership matrix.

**indicators**	**Level 1**	**Level 2**	**Level 3**	**Level 4**	**Level 5**
SA	−0.388	−0.130	0.247	−0.008	−0.528
LF/HF	0.410	−0.272	0.028	−0.012	−0.332

To comprehensively evaluate the physiological fatigue and psychological load of drivers, entropy weight method is adopted to calculate the weight of indicators (36), and the weight values of SA and LF/HF are obtained, as shown in Table 6.

**Table 6:** Index weight.

**Indicators**	**SA**	**LF/HF**
e_j_	0.9952	0.9918
w_j_	0.7143	0.2857

*EJ represents the information entropy value of evaluation index, and the larger the index is, the more information will be reflected. Wj represents the comprehensive weight of indicators*.

## Discussion

### Under Low Road Luminance

Physiological fatigue and psychological load for drivers at low road luminance were broadly similar to those reported in the literature (13, 34). The SA index selected in this paper is negatively correlated with driving time as a whole, and the fatigue degree of drivers accumulates with the increase of driving time. SA increases relatively slowly and fluctuates in the first 5 min, but increases rapidly from the 6th min. However, the selected LF/HF index increases rapidly when the driver starts to set off, so the time point at which physiological fatigue starts to intensify and accumulate is different from the time point at which psychological load starts to accumulate. It indicates that the physiological fatigue caused by long tunnel driving is different from the fatigue caused by insufficient sleep, which is in a state of lack of alertness first. At the same time, according to China tunnel lighting standard, the road luminance level of the interior zone is set according to the same standard. The standard is reasonable for the road luminance setting of the interior zone of medium and short tunnels, but the road luminance setting for the interior zone of long tunnels needs to be improved.

### Mathematical Model Evaluation

**Figure 7:**
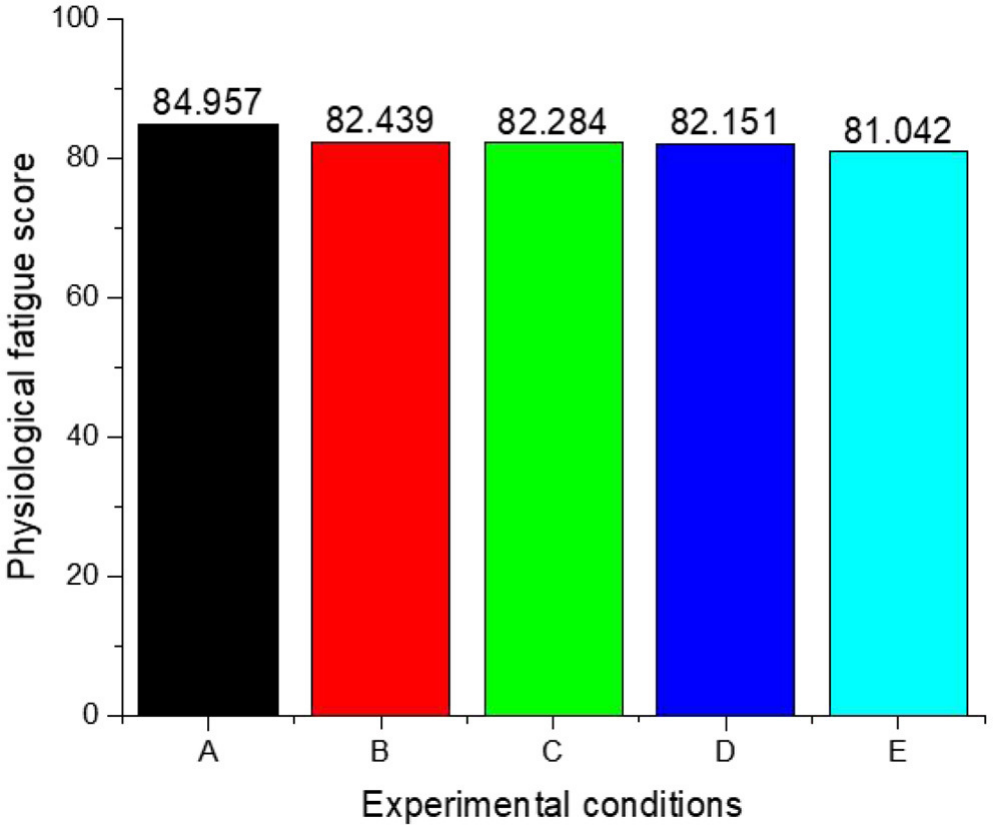
Comprehensive fatigue scores of physical fatigue and psychological load in different experimental groups.

In this paper, to comprehensively evaluate the physiological fatigue and psychological load of drivers, a fuzzy mathematical evaluation model was established according to the SA and LF/HF indexes measured in the experiment. On the basis of obtaining the membership matrix and index weight, the matrix is calculated by Python according to Equation (3).


(3) $$\begin{array}{l} t=W*R \end{array}$$

where t is the result vector of fuzzy comprehensive evaluation, t = (T1, T2..., TN), W is membership degree matrix, and R is index weight vector. After obtaining the result vectors, assign values to different fatigue degrees and define the score vector S = (50, 60, 70, 80, 90) T. The vector product of T and S is the final comprehensive score F, which represents the comprehensive fatigue scores of physiological fatigue and psychological load of the subject. The above methods were repeatedly used to calculate the comprehensive fatigue scores of physical fatigue and psychological load of the subjects under 5 different experimental groups in the interior zone of the long tunnel (hereinafter referred to as the comprehensive fatigue scores). As shown in Figure 7, the higher the comprehensive fatigue score is, the more tired the driver is, the lower the alertness is, and the higher the psychological load is.

As can be seen from Figure 7, after entering the interior zone of the long tunnel, the comprehensive fatigue score of the subjects in experimental condition A was the highest. This is consistent with the lowest road brightness and perceived environmental luminance in experimental condition A, which indicates that the comprehensive score is inversely proportional to the perceived environmental luminance. After the road luminance of group B and group C increased, the perceived environment luminance increased at the same time, and the comprehensive score decreased significantly compared with the low luminance environment, but the comprehensive score of group B and group C had few differences. This phenomenon may be caused by the fact that the experimental groups A, B, and C are all single lighting scenes and do not perceive the change of environmental luminance. The driver will still be in comprehensive fatigue after the increase of driving time in the long tunnel, but the fatigue accumulation rate of different environmental luminance is different. The above shows that the road luminance in the interior zone of the long tunnel is not the better if it is set higher. If there is no effect of auxiliary lighting setting, the road luminance setting in experimental group B can meet the comprehensive fatigue score of the driver in the long tunnel. However, the energy consumption required by experimental groups B and C to improve the road luminance is far higher than that of group A. The comprehensive fatigue scores of experimental groups D and E were better than those of A, B, and C. This indicates that without additional increase in road luminance, wall profile markers can effectively improve the physiological fatigue and psychological load of drivers and effectively save energy consumption. The higher the density of profile markers, the higher the perceived environmental luminance, and the more obvious the comprehensive improvement of fatigue degree.

### Reasons for Contour Marking to Improve Driver's Comprehensive Fatigue

Previous studies have explained the effects of adding auxiliary lighting facilities on reducing physiological fatigue of drivers from two aspects of visual comfort and non-visual effects. From the perspective of visual comfort, the addition of contour marks provides a method to improve the light environment of highway tunnel with low illumination mainly focusing on line of sight induction (39). The tunnel sidewall with high luminance can optimize the driving environment and improve the visual parameters of drivers (40). From the perspective of non-visual effects, it is generally believed that short wavelengths and light can affect the circadian system of humans (24), especially in inhibiting melatonin, thus reducing fatigue and activating body functions (41). The headlights of the vehicle used in the test were white LED with a color temperature of 6,500 K. Therefore, this paper considers that in addition to the increase of the perceptual environment brightness, the perceptual light environment composed of mixed light may also be one of the factors to improve the driver's comprehensive fatigue. The high perceived luminance level and the percentage of blue light in the mixed light under the action of contour markers produced a stimulus that alleviated driver fatigue and had a positive effect on driver alertness in long road tunnels (42, 43). Due to the difficulty of real-world driving tests, the number of subjects in this study was limited, so the influence of statistical factors such as gender and age was not taken into account. Future studies can increase the sample size and expand the experimental conditions. In addition, the design parameters of contour markers can be further optimized and improved with the help of simulation experiment scenes to reduce the comprehensive fatigue of drivers more effectively.

## Conclusion

To study the characteristics of physiological fatigue and psychological load of drivers in long tunnel, a real vehicle driving test was carried out in tunnel environments with different road brightness and enhanced tunnel environments with contour targets based on Dongyangguan Tunnel in Changzhi, Shanxi Province. Careful consideration of the test results and discussion can lead to several conclusions. They are as follows:

(1). Perceived ambient luminance is an important index to evaluate the lighting quality of the interior zone of a long tunnel. It includes the road luminance and the luminance reflected from the sidewall of the tunnel into the eyes under the irradiation of the car lamp and is related to the driving speed. However, there is only a value range for road luminance in the existing specifications, which is still defective in the physiological and psychological evaluation of drivers during driving.

(2). According to China tunnel lighting standard, in the interior zone of long tunnel with low perceived ambient luminance, the driver's comprehensive fatigue will fluctuate at first and increase rapidly after 5 min of driving. Note this standard is reasonable for the luminance settings of short and medium tunnels, but the luminance settings of long tunnels need to be improved.

(3). The road luminance of the interior zone of the long tunnel is not better if the road luminance is set higher. If there is no effect of contour mark, even if the road luminance reaches 10 cd/m^2^, its comprehensive fatigue score still reaches 82.284 points, which is higher than 82.151 and 81.042 points after setting contour mark. It indicates that the lighting environment with the perception of environmental brightness changes can effectively reduce the physiological fatigue of drivers, improve the psychological load, and save lighting energy consumption.

(4). As the setting density of contour marks in the interior zone increases, the overall fatigue of the driver is improved more quickly, and energy consumption can be effectively saved at the same time. The higher the setting density of contour markers, the higher the perceived brightness of the environment, and the more obvious the effect of comprehensively improving the degree of fatigue.

This paper provides a new idea for the lighting design of the interior zone of long tunnel by putting forward the non-visual effect of drivers' perception of ambient luminance on driving in long tunnel. That is, under the condition of not increasing the original lighting energy consumption, the traffic safety settings such as the outline sign in the tunnel can improve the driver's alertness in the long tunnel, reduce his fatigue, and reduce his psychological load. However, the subjects selected in this paper have few differences in age, and the gender difference of drivers is not taken into account, which can be further improved in the subsequent indoor simulation experiment.

## Data Availability Statement

The raw data supporting the conclusions of this article will be made available by the authors, without undue reservation.

## Author Contributions

LP was the thesis writer. JW was the thesis project provider. YY and HW respectively participated in the data and experiment work of the thesis. All authors contributed to the article and approved the submitted version.

## Funding

This study was supported by the National Natural Science Foundation of China.

## Conflict of Interest

The authors declare that the research was conducted in the absence of any commercial or financial relationships that could be construed as a potential conflict of interest.

## Publisher's Note

All claims expressed in this article are solely those of the authors and do not necessarily represent those of their affiliated organizations, or those of the publisher, the editors and the reviewers. Any product that may be evaluated in this article, or claim that may be made by its manufacturer, is not guaranteed or endorsed by the publisher.
